# Microstructure and Properties of Polyhydroxybutyrate-Chitosan-Nanohydroxyapatite Composite Scaffolds

**DOI:** 10.1100/2012/537973

**Published:** 2012-04-01

**Authors:** L. Medvecky

**Affiliations:** Department of Electroceramics, Institute of Materials Research of SAS, Watsonova 47, 040 01 Kosice, Slovakia

## Abstract

Polyhydroxybutyrate-chitosan-hydroxyapatite (PHB-CHT-HAP) composite scaffolds were prepared by the precipitation of biopolymer-nanohydroxyapatite suspensions and following lyophilisation. The propylene carbonate and acetic acid were used as the polyhydroxybutyrate and chitosan solvents, respectively. The high porous microstructure was observed in composites and the macroporosity of scaffolds (pore sizes up to 100 *μ*m) rose with the chitosan content. It was found the reduction in both the PHB melting (70°C) and thermal degradation temperatures of polyhydroxybutyrate and chitosan biopolymers in composites, which confirms the mutual ineraction between polymers and the decrease of PHB lamellar thickness. No preferential preconcentration of individual biopolymers was verified in composites, and the compressive strengths of macroporous PHB-CHT-HAP scaffolds were approximately 2.5 MPa. The high toxic fluorinated cosolvents were avoided from the preparation process.

## 1. Introduction

Composite biopolymer-calciumphosphate systems are very interesting from the point of view of the applications in reconstruction and regenerative medicine, maxillofacial surgery, and other medicine fields. The chitosan represents polysaccharides that have inductive and stimulation activity on connecticve tissue rebuilding [1]. Osteoblast-like cell growth in the calcium phosphate- (10 wt%) reinforced chitosan scaffolds was studied by Y. Zhang and M. Zhang [2]. The nanohydroxyapatite addition to chitosan improved the bioactivity of composite scaffolds and affected on the apatite formation on them [3]. The bioresorption of nanohydroxyapatite was improved, and it was assumed that it was caused the lowered migration of nanoapatite particles into surrounding tissues by the addition of chitosan [4]. The poly(3-hydroxybutyrate) (PHB) represents natural biodergadable and hydrophobic biopolymer. The porous hydroxyapatite-polyhydroxybutyrate-co-valerate scaffold (2 wt% hydroxyapatite) was prepared by the lyophilisation of suspension, and the results showed the rise in stiffness, strength, and improving in-vitro bioactivity of the scaffold [5]. The injection and compression moulding are the most utilized preparation method for the production of hydroxyapatite-polyhydroxybutyrate composites. The increase in interfacial shear strength and the enhanced endosteal bone growth were found in dry-blended and injection-moulded 30 wt% hydroxyapatite—70 wt% polyhydroxybutyrate composite [6]. In polyhydroxybutyrate-chitosan blends prepared by mixing of the individual polymer solutions dissolved in fluorinated cosolvent, 1,1,1,3,3,3-hexafluoro-2-propanol, the decrease in polyhydroxybutyrate crystallinity with chitosan content was observed. After blends melting, the miscibility of polymers was verified with the strong intermolecular interaction between polyhydroxybutyrate and chitosan chains [7, 8].

In this paper, we studied the preparation, microstructure, and properties of the polyhydroxybutyrate-chitosan-hydroxyapatite composite scaffolds using the polymer precipitation by mutual polymer solution mixing. This preparation process allows to prepare the above composite scaffolds without applying the toxic fluorinated cosolvent, whereas the propylenecarbonate and acetic acid were used as polyhydroxybutyrate (PHB) and chitosan (CHT) biopolymer solvents. Besides, our aim was to prepare relatively soft biocomposite material, which could be simply tamable to required shape.

## 2. Materials and Methods

### 2.1. Materials

Calcium-deficient nanohydroxyapatite (HAP) was synthesized by the coprecipitation of Ca(NO_3_)_2_·4H_2_O (Sigma-Aldrich, analytical grade, concentration of 0.5 mol dm^−3^) and (NH_4_)_2_HPO_4_ (Sigma-Aldrich, analytical grade, concentration of 0.5 mol dm^−3^) solutions with a molar ratio of Ca/P = 1.66. The aqueous solution of Ca^2+^ ions was slowly dropped to aqueous solution of phosphate ions during 1.5 hours. The pH was kept at 10.5 by adding of NH_3_(aq) (1 : 1). Ageing time was 72 hours. Precipitates were washed with distilled water and filtered over the membrane filter (Millipore, 0.2 *μ*m pore size). Nanohydroxyapatite (HAP) powders were dried at 110°C for 2 hours.

### 2.2. Composite Preparation

The composites with 80 wt% HAP content and various polyhydroxybutyrate (GoodFellow) to chitosan (SigmaAldrich, middle, 80% deacetylation degree) ratios (3 : 1, 1 : 1, 1 : 3) were prepared by the mutual mixing of HAP, PHB (propylenecarbonate was used as solvent), and chitosan solutions (1% acetic acid solution as solvent) in appropriate amounts. Note that the same solution volumes with different polymer concentrations were for the precipitation, and the pure PHB-HAP composite was precipitated after mixing of the suspension with acetic acid solution for satisfying similar preparation conditions. The mixing was done with a magnetic stirrer at 400 rpm. After 15 minutes, the acetone was slowly added to suspensions for the completely biopolymers precipitation. Final composites were filtered, washed with acetone, and dried at 50°C for 30 minutes, moulded to cylindrical form (6 mm D × 12 mm H), freezed at −20°C, and lyophilised (Ilshin) for 6 hour.

### 2.3. Methods

#### 2.3.1. Compressive Strength Measurements and In-Vitro Bioactivity Testing

The compressive strength of composites was measured on discs with dimensions of 6 mm in diameter and 12 mm in length. For each experimental group (5 samples), the compressive strengths were measured on a universal testing machine (LR5K Plus, Lloyd Instruments, Ltd.) at a crosshead speed of 0.5 mm/min. The in-vitro apatite-ability forming of composites was analysed from the mass increments after soaking of samples in 100 mL of simulated body fluid [9] for 1 and 2 weeks at 37°C. The SBF solutions during testing were exchanged after 3 days.

#### 2.3.2. Characterization Methods

The thermal degradation and melting of composites were analysed by the differential scanning calorimetry (DSC) and thermogravimetry (TG) (Mettler, 2000C). The phase composition and crystallinity were studied by XRD diffraction analysis (Philips $\text{X}\overset{ˇ}{\;}\text{Pert}$ Pro) using CuK*α* radiation and infrared spectroscopy (Specord M80). The microstructure of composite scaffolds was observed by a scanning electron microscopy (FE SEM JEOL7000), and the HAP particle morphology was analysed using transmission electron microscopy (TEM, TESLA BS500). The optical fluorescence microscopy (inverted optical microscope Leica DM IL LED) with blue filter was used for verification of the distribution of individual biopolymers in composites whereas the 0.1% Nile red (acetone solution, prepared from the Nile blue A according to Greenspan et al. [10]) and 0.1% eosin Y (methanol solution) [11] were applied for the detection of PHB and chitosan, respectively. The HAP-specific surface was determined by the N_2_ adsorption method at −196°C (GEMINI).

## 3. Results and Discussion

### 3.1. Microstructure Analysis of Composite Scaffolds

**Figure 1:**
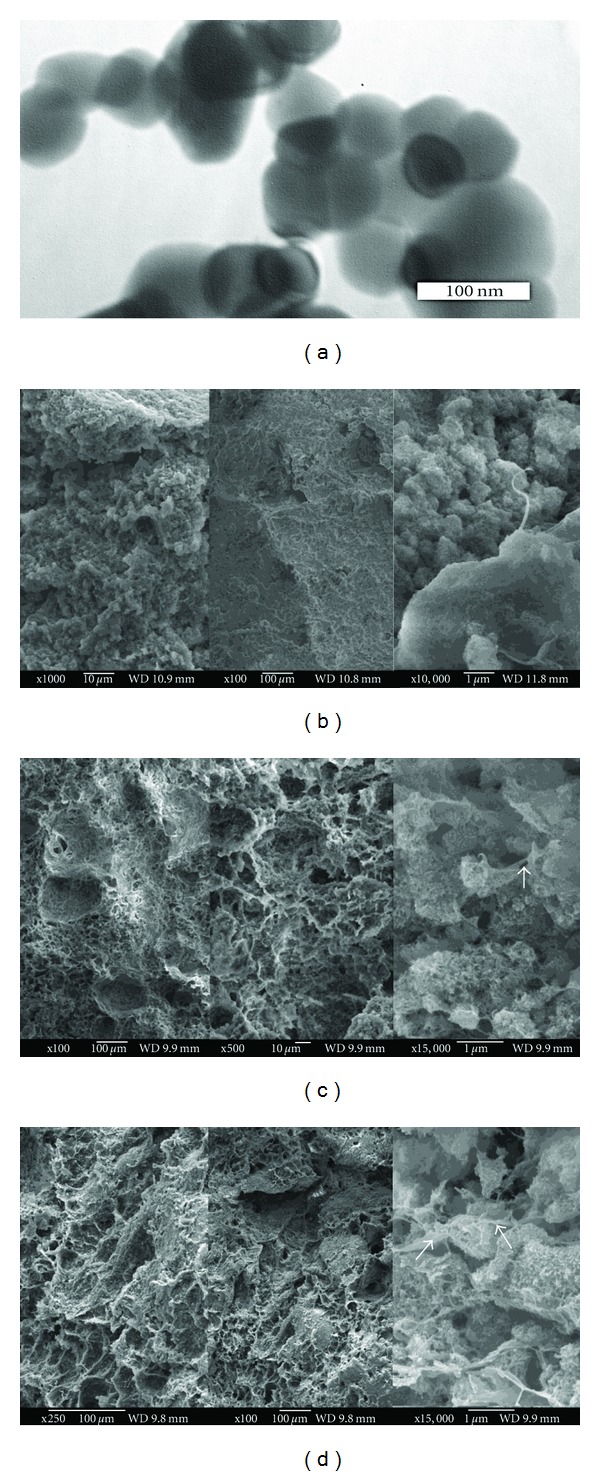
Morphology of HAP particles (a) and microstructure of composite scaffolds with different PHB : CHT ratio: (b) 3 : 1; (c) 1 : 1; (d) 1 : 3 (arrows show biopolymer fibre connections of agglomerates).

**Figure 2:**
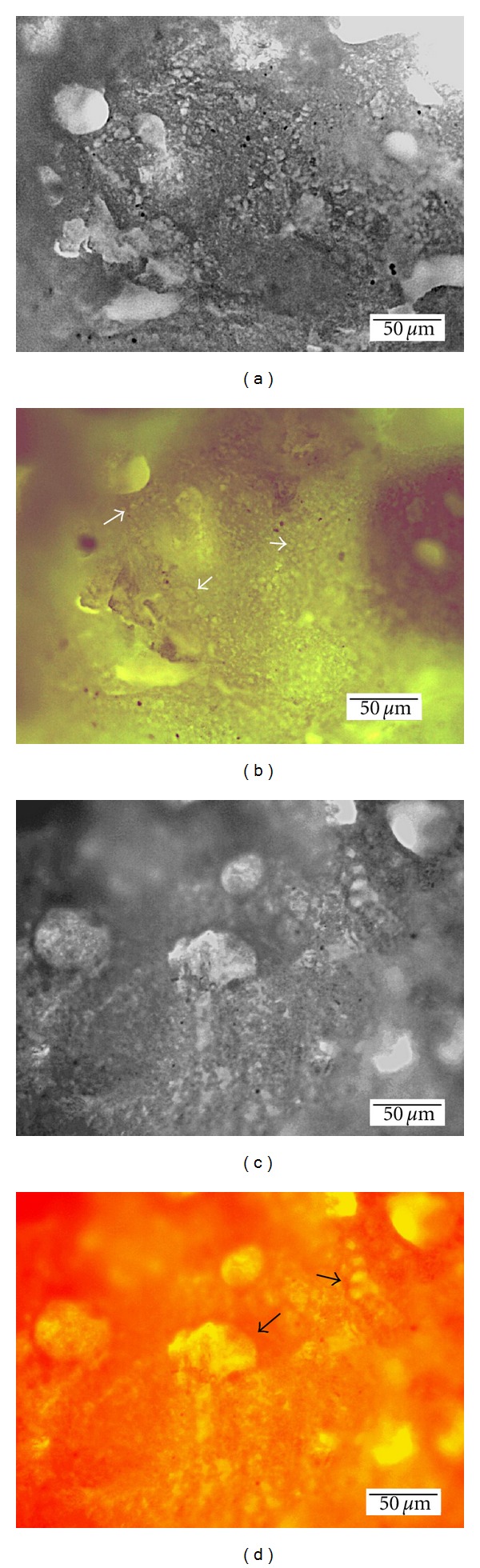
Optical micrographs of composite scaffolds with PHB : CHT = 1 : 1 in transmitted (a, c) and fluorescence mode (b) eosin Y (chitosan marker), (d) Nile red (PHB marker) (arrows show pore walls).

The HAP particles had spherical morphology with the average particle size around 50 nm (Figure 1(a)). The value of specific surface was 96 m^2^/g, from which results the average particle size (for spherical particle shape approximation) equals 22 nm. This value is comparable with one calculated from the XRD patterns. The SEM microstructures of composite scaffolds with various PHB : CHT ratios are shown in Figures 1(b), 1(c), and 1(d). The composite with PHB : CHT = 3 : 1 (Figure 1(b)) had more compact microstructure, where a very small fraction of large 100 *μ*m pores and the high amount of irregular pores with dimension <10 *μ*m can be observed. Despite increased amount of the large spherical-shaped pores with chitosan content in composites, the high amount of pores with average size of 10–20 *μ*m was found in microstructures (Figures 1(c) and 1(d)). From the analysis of micrographs result that the microstructures of all samples are very porous and the micropores with dimensions less than 1 *μ*m are clearly visible too. Microstructure analysis verified that the main factor responsible for a large pores formation is the presence of sufficient fraction of the chitosan gel, in which is entrapped the high amount of water and this swelled gel has significantly enlarged volume than native chitosan powder. Besides, the chitosan supports the formation longer fibres or plates in composites and stabilizes walls of pores whereas they are not visible in composite with lower chitosan content. It can be observed in Figures 1(b)–1(d) that composite agglomerates are mutually interconnected via biopolymer fibres, which stabilizes the composite scaffold microstructure. Note that the measured composite scaffold densities were maximal at PHB : CHT = 1 : 1 (0.45 g/cm^−1^) and the porosities were about 80 vol%. Thin (<50 nm) needle-like particles or dendrites were found in all microstructures whereas the highest width/length ratio was found in composite with PHB : CHT = 1 : 1. The distribution of individual biopolymers in composite with PHB : CHT = 1 : 1 was analysed by the fluorescence of biopolymers after interaction with the specific dye (Figure 2). The thin sample plates were cut from fractured surfaces of composite samples. From the comparison of fluorescence images results, both biopolymers are homogeneously distributed in composite microstructures without preferential preconcentration in any location. In these images is clearly visible more intensive fluorescence from chitosan (green to brown colour in colour image) and PHB (orange—red colour in colour image) in pore walls, where is naturally the higher concentration of composite (and biopolymers). Thus, composite fibres or plates, which form the pore walls, contain both biopolymers.

### 3.2. XRD and IR Analysis of Composites

**Figure 3:**
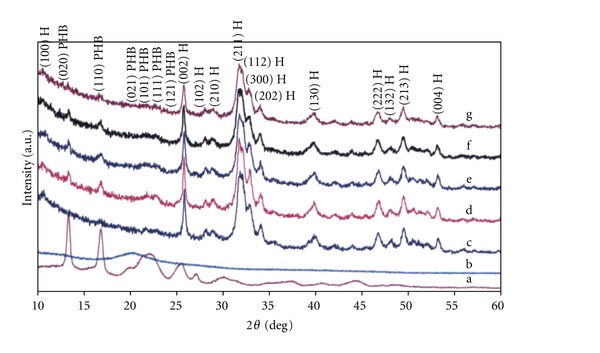
XRD patterns of composites with different PHB : CHT ratio. ((a) pure PHB; (b) pure chitosan; (c) pure HAP; (d) PHB : CHT = 3 : 1; (e) 1 : 1; (f) 1 : 3; (g) 0 : 1). (H: hydroxyapatite lines).

The XRD pattern of HAP verifies the presence of nanoapatite-like phase (JCPDS 24-0033), and the crystallinity size calculated from the reflections of (002) hydroxyapatite plane using the Scherrer equation was about 30 nm. No significant changes were observed in the XRD composite patterns (Figure 3) at different PHB : CHT ratios. Besides, reflections from HAP planes and reflections from (020) and (110) PHB planes were clearly visible in the XRD patterns, which confirms the presence of significant PHB fraction in crystalline state. The CHT precipitated mainly in amorphous state. Very small increase of the HAP peak widths in composites was found only that corresponds with partially HAP particle dissolution in weak acid suspensions. 

**Figure 4:**
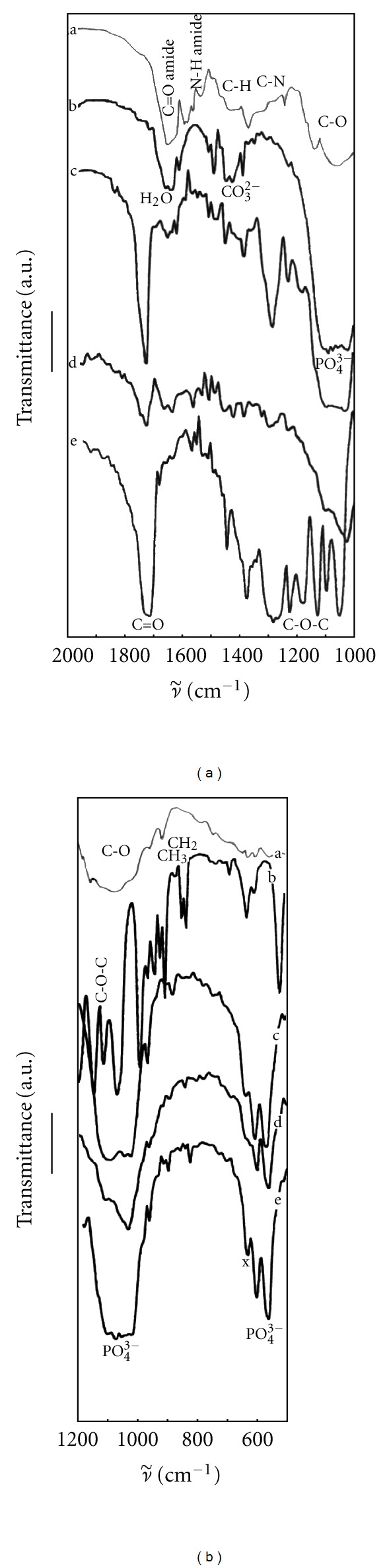
IR spectra of composites and pure components. (a), curve a and (b) curve a: pure chitosan; (a) curve b and (b) curve c: pure HAP; (a) curve c and (b) curve e: PHB-HAP composite; (a) curve d and (b) curve d: composite with PHB : CHT = 1 : 3; (a) curve e and (b) curve b: pure PHB) (x-bending P–OH in hydroxyapatite).

The IR spectra of composites are compared in Figure 4. The characteristic vibration of PO_4_^3−^ group located at 1050, 1100, and 962 cm^−1^ (antisymmetric (*ν*_3_) and symmetric (*ν*_1_) P–O stretching vibrations) and O–P–O bending (*ν*_4_) vibrations at 565 and 603 cm^−1^ can be found in HAP (Figure 3, curve (a)) [12]. Also, *ν*_2_ and *ν*_3_ modes of CO_3_^2−^ are located at wave numbers of 870 and 1400–1550 cm^−1^, the librational mode of OH hydroxyapatite group at 630 cm^−1^ [13], the broad band around 1650 cm^−1^ indicate adsorbed H_2_O (Figures 4(a), curve (b) and 4(b), curve (c)). From the detailed analysis of carbonate bands, it results that the AB type of carbonated HAP is formed by the CO_2_ adsorption from air because peaks 1450 cm^−1^, 1420 cm^−1^, and 1550 cm^−1^ were found in spectrum. In the spectra of PHB-HAP composite (Figures 4(a) curve (c) and 4(b) curve (e)), besides HAP bands, the band from C=O stretching vibration (from PHB) at 1725 with shoulder at 1750 cm^−1^, bands at 1280, 1230, and 1180 cm^−1^, ascribed to the stretching vibrations of the C–O–C ester groups and bending CH_2_-, CH_3_-group vibrations under 1000 cm^−1^ were observable [14]. No shifts in the peak locations of carbonyl and ester bonds vibrations were found but the peak at 870 cm^−1^ from CO_3_^2−^ vibration in HAP vanished (bands at 1400–1550 cm^−1^ are overlapped with PHB vibration peaks) from spectra. In the case of three component systems (Figure 4(a) curve (d) and 4(b), curve (d)), the chitosan (Figures 4(a) curve (a), and 4(b) curve (a)) amide I C=O vibration band at 1650 cm^−1^ cannot be clearly resolved in spectrum because of its overlapping with the band of physisorbed H_2_O, the amide II N–H deformation vibration bands at 1550 cm^−1^, and band around 1050 cm^−1^ corresponding to stretching C–O vibrations which are visible in spectrum [15, 16]. No shift was observed in stretching vibrations of the PHB carbonyl or C–O ester group after chitosan mixing. The intensity of PHB carbonyl vibration in composites at 1750 cm^−1^ significantly increased in comparison with the peak at 1725 cm^−1^.

### 3.3. TG and DSC Measurements, Mechanical Properties and In-Vitro Bioactivity of Composites

**Figure 5:**
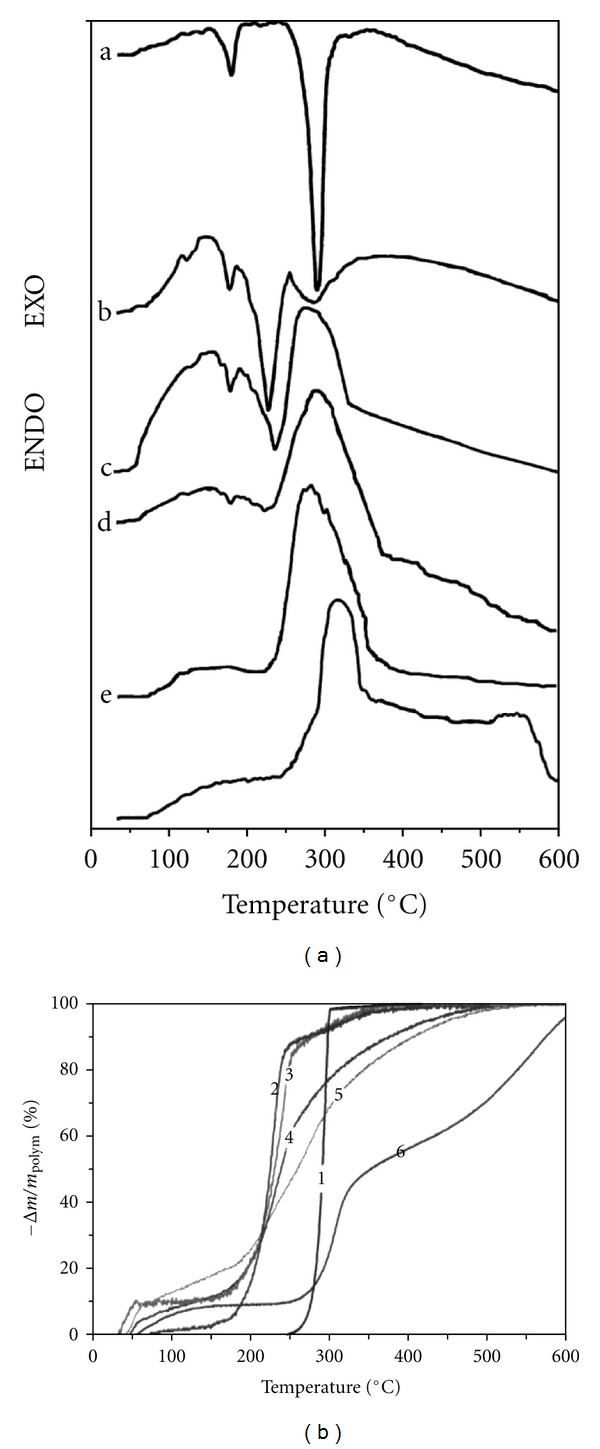
DSC (a) (a: pure PHB; b: PHB-HAP composite; c: composite with PHB : CHT = 3 : 1; d: composite with PHB : CHT = 1 : 1; e: composite with PHB : CHT = 1 : 3; f: pure chitosan) and TG ((b) *y*-axis represents the ratio of mass losse to total biopolymer content in composite) (curve 1: pure PHB; curve 2: PHB-HAP composite; curve 3: composite with PHB : CHT = 3 : 1; curve 4: composite with PHB : CHT = 1 : 1; curve 5: composite with PHB : CHT = 1 : 3; curve 6: pure chitosan) analysis of composites.

Results of TG and DSC measurements of samples are shown in Figure 5. Two characteristic endoeffects with maxima at 182 and 292°C were found on the DSC curve of PHB (Figure 5(a), curve a), which corresponds to melting temperature and the PHB decomposition with rapid mass losses on TG curve (Figure 5(b), curve 1). Three endoeffects at 181, 230, and 290°C are visible on PHB-HAP composite DSC curve whereas approximately 90% of PHB amount decomposed at 230°C and 10% in the second stage of decomposition at 290°C (Figures 5(a), curve b, and 5(b) curve 2). After addition of third component (chitosan) to composite, three endoeffects at 163, 181 (PHB melting), and 238°C from the PHB decomposition and single wide exoeffect at 290°C were observed (Figures 5(a) curve c, and 5(b) curve 3). The exoeffect represents the chitosan thermal decomposition, where the peak integral intensity rose with the chitosan content in composites. Besides, it can be observable the small temperature shift to lower temperature or the presence of shoulder on low temperature peak side as the consequence of the addition of both the chitosan exo- and PHB endoeffects (at 230°C). The pure chitosan (Figures 5(a), curve f, and 5(b) curve 6) decomposes in three steps as it can be visible from TG curve—water release up to 150°C, the weight loss between 200 and 300°C may be related to the amine units decomposition, saccharide units are degraded above 300°C and decomposition finished around 600°C [17]. On the chitosan DSC curve, two large exoeffects are observable—at 310 and 550°C with “exoplateau” among peaks. From the comparison of TG curves of composites, it results that they shift slowly to higher temperatures with the chitosan content but curves are smooth and the single inflex point was found after their numerical differentiation only. 

The IR spectroscopy verified changes in crystallinity of PHB, where the increase in intensity of peak at 1750 cm^−1^ was observed after the chitosan addition, and the intermolecular bonding between biopolymers was not confirmed. This peak was also present in the carbonyl vibration band of PHB-HAP composite but with lower intensity and it corresponds to rise of the amorphous fraction in PHB [14]. Ikejima et al. [15] confirmed by ^13^C NMR spectroscopy the intermolecular bonding between the PHB carbonyl and chitosan amide groups and showed the increase of amorphous PHB phase component with chitosan content in the PHB-chitosan composites. Besides, on the DSC curves of these composites, the low temperature PHB melting point at 160°C and the increase of endoeffect intensity at this temperature with chitosan content were found, which was caused by the miscibility of amorphous PHB phase and chitosan. Similar dependence of the PHB melting point depression was found by Cheung et al. [7] and the intermolecular interaction between biopolymers was verified using ^1^H NMR spectroscopy. Ikejima and Inoue [8] showed that the suppress of the PHB melting point in the blends is caused by the decrease in lamellar thickness of the PHB crystallites, and the rigid chitosan molecules make PHB molecules in the blends inflexible and suppress the crystallization of PHB. Sudesh et al. [18] found variation in melting temperature as a function of the inverse lamellar thickness for melt-crystallized PHB and melting point reduction from 180 to 160°C correspond with the decrease in lamellar thickness from 10 nm to 5 nm. These facts clearly showed that the endoeffect at 163°C represents melting of the amorphous PHB formed after chitosan addition to the PHB-CHT-HAP composites. The amorphous PHB in composites creates, as a result of miscibility, mutual interaction between biopolymers and PHB lamellar thickness decrease despite the different preparation procedure and miscellanous solvents used for biopolymer dissolution.

The thermal decomposition of both biopolymers in composites was strongly affected by the addition of nanohydroxyapatite. The PHB thermal decomposition in PHB-HAP composite was shifted about 70°C to lower temperature in comparison with pure PHB. Chen and Wang [19] showed approximately 20°C depress in the degradation temperature after 30 wt% addition of HAP to polyhydroxybutyrate—co-valerate biopolymer (composite was prepared by compression moulding). Misra et al. [20] found similar shift in the PHB decomposition temperature (from 290 to 230°C) in PHB-bioglass composite foams. Kim et al. [21] verify that Ca^2+^ ions enhance and catalyse the depolymerisation of PHB molecules in very low concentration and reduce thermal decomposition temperature. The calcium ions act as Lewis acid that interacts with carboxyl group facilitating the formation of the double bond in crotonyl unit. Csomorová et al. [22] showed that the degradation temperature was influenced even in powder PHB-CaO mixtures. We believe that in the case of composites with HAP addition, the same effect of calcium ions was manifested because HAP has a positively surface zeta potential in acid and weak alkaline solutions [23]. From the TG and DSC analysis, it clearly results that the mechanism of chitosan thermal degradation was significantly changed in composite systems, where the multistage degradation mechanism with distinct thermal steps was changed to almost single stage one. The degradation was finished at about 200°C lower temperature than in the pure chitosan. This facts could be the result of biopolymer interaction of the surrounding hydrophilic chitosan chains with hydrophobic PHB macromolecules [8], which make impossible to connect chitosan chains into larger structures and weaken intermolecular interactions. In the microstructures, were found the fine crystallities or dendrities formed during the crystallization of PHB polymer, which contains nanohydroxyapatite. The formation of needle-like PHB crystallites from propylene carbonate, observed by Organ et al. [24] and Iwata et al. [25] during the precipitation of PHB, covalerates copolymer from chloroform-ethanol solutions. 

**Figure 6:**
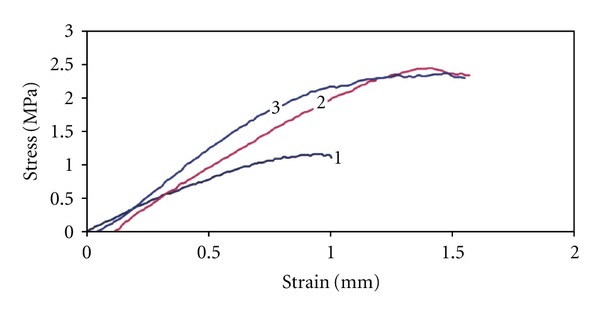
Stress-strain curves of composite scaffolds with different PHB : CHT ratio. (curve (1) 3 : 1; curve (2) 1 : 1; curve (3) 1 : 3).

The compressive strength (CSs) of composite scaffolds rose with the chitosan content, and they equal to 1.1 ± 0.2, 2.3 ± 0.2, and 2.5 ± 0.3 MPa for composites with PHB : CHT = 3 : 1, 1 : 1, 1 : 3. The PHB-HAP composite disintegrated under weak loading. From the comparison of typical composite scaffold stress-strain curves (Figure 6), results rise in the plasticity of composites with the chitosan content because of the higher fraction of larger pores in microstructure. The gradual reduction of the composite scaffold CSs (no significant differences between composites with PHB : CHT = 1 : 1, 1 : 3) to 1.8 ± 0.2 and 1.6 ± 0.3 MPa after SBF soaking for 1 and 2 weeks, respectively, was found, which confirms slow degradation of the biopolymer matrix in SBF. Li et al. [26] synthesized nanohydroxyapatite-chitosan composite by the coprecipitation method, moulded in clava for obtaining dense microstructure, and CSs were around 100 MPa. Y. Zhang and M. Zhang [27] prepared the CHT-*β* tricalcium phosphate composites with 80 vol% porosities by the lyophilisation of suspension and they had very low CS (around 0.3 MPa). The CSs of porous hydroxyapatite or calcium phosphate ceramics (80% porosity) were low and they do not exceed 0.4 MPa [28]. In the wollastonite-polyhydroxybutyrate-co-valerate composite with 60 wt% of wollastonite and high porous microstructure, CS equals 0.28 MPa [19]. Prepared biocomposites are soft and the mutual interconnections of individual composite components are sufficient for manipulation and mechanical treatment like cutting with scalpel. 

Results of the in-vitro apatite-ability forming of composites (Table 1) showed that the mass increments in composites were independent on biopolymer ratio, where 17–20 mass % increments were observed after 2 weeks soaking in SBF. This fact is understandable because the contents of hydroxyapatite bioactive component in composites were the same. Note that none mass losses verified the slow degradation of biopolymers during soaking only.

## 4. Conclusion

The results of experimental work can be summarized in the following points.

1. High porous microstructure was observed in composite scaffolds and the macroporosity of scaffolds rose with the chitosan content in composites.
2. Rise in amount of the amorphous PHB component was found after chitosan addition to the PHB-HAP mixture.
3. Reductions in both the PHB melting and thermal degradation temperatures of PHB and chitosan biopolymers in composites were noted, which confirms mutual ineraction between polymers and the decrease of PHB lamellar thickness.
4. Biopolymers were homogeneously distributed in composite scaffold microstructures.
5. Compressive strengths of macroporous PHB-CHT-HAP scaffolds were approximately 2.5 MPa.
6. High nanohydroxyapatite loading of biopolymer matrix was achieved, wich preserves the appropriate in-vitro apatite-ability forming of composites.
7. Composite scaffolds were prepared without applying of the high toxic fluorinated co-solvents.

## Figures and Tables

**Table 1:** The changes in masses of composites after soaking in SBF for 1 and 2 weeks.

Soaking Time/week	Mass increments/mass %		
	80%HAP-20% (25%CHT + 75%PHB)	80%HAP-20% (50%CHT + 50%PHB)	80%HAP-20% (75%CHT + 25%PHB)
1	13.2 ± 0.8	14.1 ± 0.9	12.3 ± 1.1
2	17.8 ± 1	16.7 ± 0.8	20 ± 1.5
